# Case report: Obstructive jaundice caused by biliary cystadenoma

**DOI:** 10.3389/fonc.2023.1165979

**Published:** 2023-03-30

**Authors:** Yun-cheng Li, An-da Shi, Kang-shuai Li

**Affiliations:** Department of General Surgery, Qilu Hospital, Cheeloo College of Medicine, Shandong University, Jinan, China

**Keywords:** hepatic cyst, biliary cystadenoma, obstructive jaundice, endoscopic nasobiliary drainage, common bile duct (CBD)

## Abstract

Biliary cystadenoma (also called mucinous cystic neoplasm with low-grade intraepithelial neoplasia) is a rare cystic tumor that arises from the biliary epithelium. The cause of biliary cystadenoma is still unclear. Jaundice is a rare presentation of intrahepatic biliary cystadenoma, which can lead to a diagnostic dilemma. Herein, we present a case of intrahepatic biliary cystadenoma that primarily exhibited as jaundice. A 56-year-old woman has suffered from yellow staining of her skin and sclera for more than 1 month. She had a poor appetite and mild epigastric pain. Laboratory examination showed elevated levels of total bilirubin and elevated carbohydrate antigen 19-9 (CA19-9). A contrast-enhanced computed tomography of the abdomen showed a 7.4 * 5.3-cm, oval, low-density lesion in the left liver parenchyma with a clear boundary and visible septa. The common bile duct was obviously dilated with wall thickening. On magnetic resonance imaging, the lesion in the liver showed a multilocular cystic, unenhanced long T2 signal. There was local thickening of the common bile duct wall with short T2-like filling defects and high signal intensity on diffusion-weighted imaging (DWI). The patient had no history of other malignant tumors and adjuvant therapy such as radiotherapy and chemotherapy. She was clinically suspected of having either biliary cystadenoma or a malignancy; hence, resection was performed. Macroscopically, the excised tissue specimen showed a polypoid mass in the common bile duct, which extended along the bile duct to the intrahepatic bile duct. There was a cystic and solid mass in the left liver with yellow turbid fluid, which was associated with the polypoid mass in the common bile duct. Histopathology suggests mucinous cystadenoma of the liver and hilar bile duct. The differential diagnosis of biliary cystadenoma and treatment selection have been discussed.

## Introduction

Obstructive jaundice is a heterogeneous disease characterized by bile duct obstruction and cholestasis. Patients may have typical clinical manifestations such as jaundice of the skin and sclera, dark yellow urine, and lighter or even clay-white stool. It is vital to determine the nature of the lesion for the formulation of the treatment plan. However, the differential diagnosis of obstructive jaundice is usually difficult.

Biliary cystadenoma is a rare neoplasm arising from intrahepatic or extrahepatic bile ducts. The majority of biliary cystadenoma arises from the intrahepatic ducts and is usually asymptomatic. The presenting symptoms are usually right hypochondrium pain or abdominal mass. Extrahepatic biliary cystadenoma usually presents with obstructive jaundice either directly by tumor blockage or by mucin secretion. In this paper, we present a patient with biliary cystadenoma involving the left intrahepatic and left main hepatic ducts presenting as obstructive jaundice. We also discussed the differential diagnosis and treatment selection of biliary cystadenoma.

## Case presentation

**Figure 1:**
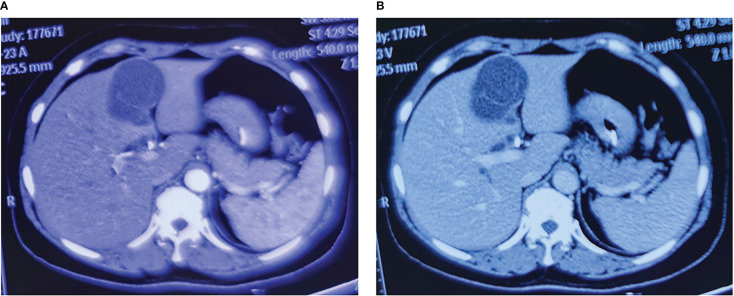
Typical images of contrast-enhanced computed tomography of the liver lesions. **(A)** The arterial phase image of the liver lesions. **(B)** The venous phase image of the liver lesions.

**Figure 2:**
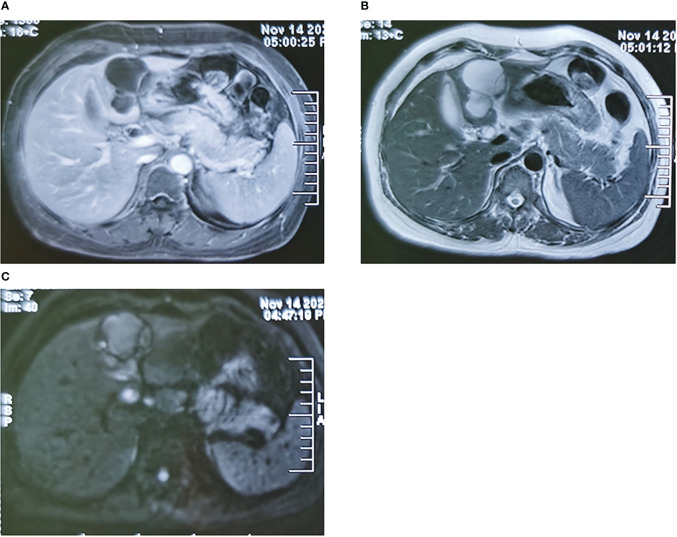
Typical images of magnetic resonance imaging of the liver lesions. **(A)** The arterial phase image of the liver lesions. **(B)** The T2 phase image of the liver lesions. **(C)** The DWI phase image of the liver lesions. DWI, diffusion-weighted imaging.

A 56-year-old woman was admitted to our hospital because of yellow staining of her skin and sclera for more than 1 month. She had received endoscopic nasobiliary drainage at a local hospital 1 day prior. She had a poor appetite and mild epigastric pain and denied a history of fever, cough, malaise, vomiting, diarrhea, or weight loss. She had no history of hepatitis B/C infection and no history of alcohol intake. Physical examination revealed jaundice, scleral icterus, and mild subxiphoid tenderness without rebound or muscle tension, and there were no signs of hepatomegaly, splenomegaly, or ascites. Relevant laboratory examination showed abnormal liver function test results, including elevated levels of total bilirubin (149.1 μmol/L; normal range, 5.0–21.0 μmol/L), direct bilirubin (118.0 μmol/L; normal range, <6.0 μmol/L), indirect bilirubin (31.1 μmol/L; normal range, 2.0–15.0 μmol/L), alanine aminotransferase (56 U/L; normal range, 7–40 U/L), aspartate aminotransferase (36 U/L; reference range, 13–35 U/L), gamma-glutamyl transferase (324 U/L; reference range, 7–45 U/L), and alkaline phosphatase (529 U/L; reference range, 50–135 U/L). A blood cell routine showed a decreased red blood cell count (2.9 * 10^12^/L; reference range, 3.8 * 10^12^/L to 5.1 * 10^12^/L) and a decreased hemoglobin (92.0 g/L; reference range, 115–150 g/L). Autoimmunological test and IgG4 were normal. Hepatitis B serologies demonstrated negative hepatitis B surface antigens and hepatitis C virus antibodies. Serum tumor marker examination showed elevated carbohydrate antigen 19-9 (CA19-9) (189.0 U/ml; reference range, 0.0–34.0 U/ml), while CA125 and carcinoembryonic antigen were within the normal range. A contrast-enhanced computed tomography of the abdomen showed a 7.4 * 5.3-cm, oval, low-density lesion in the left liver parenchyma with a clear boundary and visible septa. The septation was slightly enhanced, and there was no enhancement of its internal fluid components. The intrahepatic bile ducts were mildly dilated. The common bile duct was obviously dilated with wall thickening. The gallbladder was normal in size, but the wall was thickened. In addition, slightly larger retroperitoneal lymph nodes and cystic shadows in the duodenum could be seen. No obvious discontinuity or filling defect was found in the portal vein branch and trunk, and the presence of a cancer thrombus was not considered (**Figure 1**). On magnetic resonance imaging, the lesion in the liver showed a multilocular cystic, unenhanced long T2 signal. There was local thickening of the common bile duct wall with short T2-like filling defects and high signal intensity on diffusion-weighted imaging (DWI). The wall of the gallbladder was thickened and markedly enhanced, and several slightly larger lymph nodes were seen in the hilar region (**Figure 2**). The tube placed due to endoscopic nasobiliary drainage was visible on both CT and MRI.

One year ago, she underwent inferior vena cava filter implantation and received thrombolytic drugs because of a pulmonary embolism. At present, the filter has been removed, and thrombolytic drugs have not been taken again. Lower extremity vascular ultrasound examination showed bilateral varicose great saphenous veins and old thrombosis in the right popliteal vein and posterior tibial vein.

**Figure 3:**
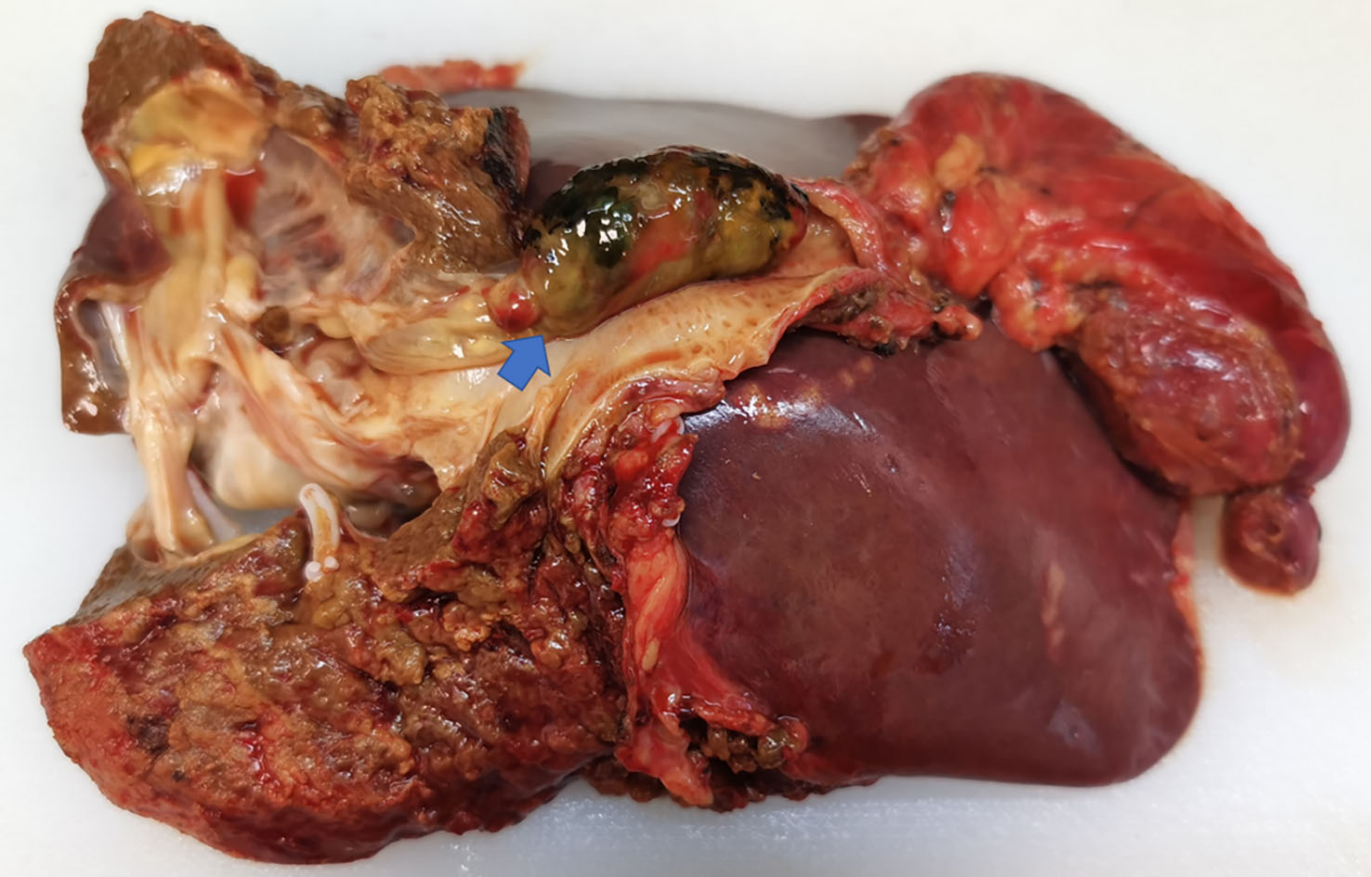
Macroscopic image of the specimen with the tumor within the common bile duct.

**Figure 4:**
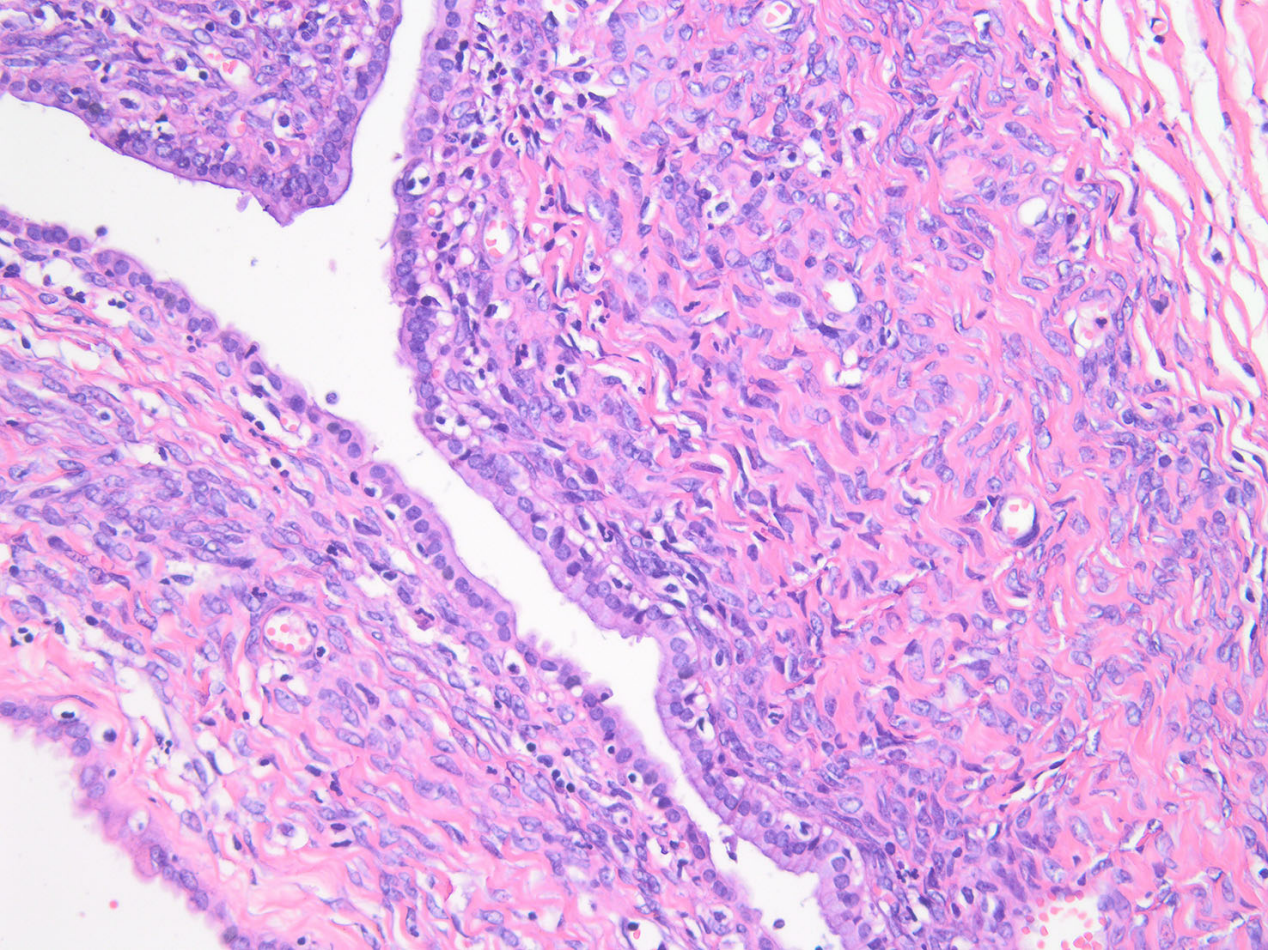
Microscopic examination of the resected specimen showing mucinous cystic neoplasm with low-grade intraepithelial neoplasia.

Preoperative ultrasound, CT, MRI, and biopsy results should be combined to determine the surgical plan; lobectomy is the preferred treatment; cystectomy is recommended only when complete resection is likely to injure the vascular structure of the liver. The patient received surgical resection after adequate preoperative preparation. During the operation, multiple cystic solid masses were palpable in the left inner lobe of the liver, cord masses were palpable in the common bile duct, and multiple enlarged lymph nodes were observed in the hepatoduodenal ligament. Considering the possibility of a left intrahepatic cystic tumor and common bile duct tumor, left hemihepatectomy, extrahepatic bile duct resection, cholecystectomy, and choledochojejunostomy were performed. The excised tissue specimen showed a polypoid mass in the common bile duct, which extended along the bile duct to the intrahepatic bile duct, and part of it was suspected to be necrotic (**Figure 3**). There was a cystic and solid mass in the left liver with yellow turbid fluid, which was associated with the polypoid mass in the common bile duct. Histopathology suggests mucinous cystadenoma of the liver and hilar bile duct (**Figure 4**). Finally, we reached a pathological diagnosis of mucinous cystic neoplasm with low-grade intraepithelial neoplasia (Biliary cystadenoma). Moreover, after surgical resection, the patient’s jaundice symptoms improved significantly, and the levels of total bilirubin decreased. The patient was discharged after stable condition.

## Discussion

In our case, the patient presented increasing jaundice with decreased appetite and mild abdominal pain. Abnormal liver function with mainly elevated direct bilirubin suggested biliary obstruction. He had a history of pulmonary embolism, and venous ultrasound of the lower extremities showed no active thrombus. Physical examination revealed mild tenderness in the upper abdomen and no rebound pain or muscle tension. CT revealed the presence of a multilocular hypodense mass in the liver with a mild enhancement of the internal septation and no significant enhancement of the fluid component. There was mild dilatation of the intrahepatic bile ducts and marked dilatation of the hilar common bile duct with thickening of the bile duct wall. Magnetic resonance imaging showed local thickening of the common bile duct and high signal on DWI. From the patient’s clinical presentation, physical examination, and imaging findings, combined with the patient’s age and sex, we considered the lesion as the following possibilities:

1. Cyst of the liver: large hepatic cysts may present with non-specific symptoms such as upper abdominal discomfort and loss of appetite. A small number of patients may have jaundice due to the compression of the bile duct by the cyst. CT plain scan showed single or multiple low-density masses in the liver, and a contrast-enhanced scan showed low-density masses without obvious enhancement. On MRI, the cyst showed a low signal on T1WI and a high signal on T2WI. Therefore, the possibility of a hepatic cyst could not be completely excluded.
2. Cholangiocarcinoma: the patient presented with progressive jaundice, CT scan showed mild dilatation of the intrahepatic bile duct and thickening and obvious dilatation of the common bile duct wall. MRI showed thickening of the common bile duct wall in the hilar region with a high signal on DWI, and the patient’s CA199 increased. It is necessary to obtain pathological results to exclude this disease.
3. Intraductal papillary neoplasm of the bile duct (IPNB): this kind of patient can present with non-specific clinical manifestations such as abdominal pain and jaundice. This disease can be manifested as hepatic cystic lesion and dilatation of the intrahepatic bile duct and common bile duct on CT; its clinical manifestations, laboratory examination, and imaging are not specific. The diagnosis of this disease mainly depends on postoperative pathological examination.
4. Biliary cystadenoma: the patient was a middle-aged woman with progressive jaundice. CT showed a well-defined, low-density intrahepatic mass with internal septa, which showed mild enhancement on contrast-enhanced CT. MRI showed a well-defined multilocular high signal occupation with internal septa on T2WI. Mucinous cystic neoplasm often occurs in the liver. According to the principle of monism, we considered that the mass of the hilar may be caused by partial detachment of the intrahepatic mass, thus causing obstructive jaundice.

Cyst of the liver is a common benign disease and generally will not exhibit clinical symptoms, and very few people will be jaundiced due to cyst compression of the bile duct. The most common clinical type is a congenital hepatic cyst, which can be divided into two subtypes: single and multiple. Multiple hepatic cysts can be seen in the liver with multiple dark areas of fluid, and several cysts may be fused to communicate or appear as several septations within a large cyst. In addition, the congenital polycystic liver is often accompanied by cysts in other organs such as the kidneys and lungs. This patient does not exhibit the typical clinical features of a cyst of the liver.

Cholangiocarcinoma is a diverse group of malignancies emerging from the biliary epithelium. According to its anatomical site, cholangiocarcinoma can be divided into intrahepatic cholangiocarcinoma (iCCA), perihilar cholangiocarcinoma (pCCA), and distal cholangiocarcinoma (dCCA). They have different clinical features. The most common symptoms of iCCA patients are right upper abdominal pain and weight loss. Jaundice occurs in approximately 25% of patients, and CA199 levels can be elevated in some patients. iCCA appears as a focal hepatic mass on CT and MRI, and the bile duct surrounding the mass may be dilated. The typical finding on an enhanced scan is that the mass has peripheral or central enhancement. Patients with pCCA may have obvious jaundice. The lesion shows delayed enhancement on CT. The characteristic is helpful for the differential diagnosis of pCCA. Histopathology is the gold standard to determine the diagnosis of cholangiocarcinoma.

Intraductal papilloma neoplasm of the bile duct is divided into intraductal papillary mucinous neoplasm of the bile duct (IPMN-B) and IPNB-without mucin secretion (IPNB-NM) according to the presence or absence of mucous secretion. While IPMN-B has similar histological features to the corresponding pancreatic lesions, IPNB-NM may represent a distinct type. IPMN-B communicates with the bile duct; mucus or tumor debris can cause intermittent bile duct obstruction, thereby causing jaundice. As the bile duct pressure rises, the mucus can be discharged, and the obstruction is relieved. Thus, jaundice is often fluctuating. Secondary dilatation of the duodenal papilla has been observed in some patients. The presence of extensive bile duct dilatation with or without tumor on imaging and mucus outflow from the opening of the duodenal papilla under endoscopic retrograde cholangiopancreatography (ERCP) are highly suggestive of this disease. Compared with IPMN-B, IPNB-NM has no mucus secretion, the downstream biliary obstruction usually does not dilate, and the degree of biliary dilation is not as obvious as that of IPMN-B. Compared with IPMN-B, IPNB-NM is more aggressive and has a worse prognosis. IPNB may have different imaging findings according to the location of the tumor, the size and morphology of the mass, and the degree of mucin secretion (1).

Biliary cystadenoma is a rare cystic tumor that tends to be more common in middle-aged women. This study reports an intrahepatic biliary cystadenoma partially shedding into the common bile duct, causing obstructive jaundice. Biliary cystadenoma mostly occurs in the liver and is often found due to non-specific abdominal pain or jaundice. On CT, it can appear as a low-density mass in the liver with internal septa and wall nodules, and enhancement can be seen along its wall and internal septum. MRI usually shows multilocular hyperintensity on T2WI with a well-defined internal septum and variable signal intensity on T1WI. Increased thickening and enhancement of the septa, nodules, and capsule with coarse calcifications have been cited as determinants of malignancy (2). The differential diagnosis of biliary cystadenoma and IPMN is difficult. Biliary cystadenoma does not communicate with the bile duct, and the mucus does not enter the bile duct. Therefore, the bile duct does not dilate, and there is no mucous outflow from the duodenal papilla. In this case, the patient had bile duct dilatation due to the obstruction of the common bile duct caused by partial tumor detachment. Pathology is the gold standard for diagnosis. In a pathological examination, the presence of ovarium-like subepithelial stroma is a tumor-specific indicator and suggests a good prognosis.

Biliary cystadenoma is a mucinous cystic tumor with malignant transformation potential. In the past, its treatment methods included aspiration, marsupialization, internal drainage, and partial excision (3). Accumulated evidence indicates that complete excision of the mass is the ideal treatment for this disease. After adequate preoperative preparation, we performed surgical treatment on the patient. Rapid pathological examination was performed during the operation to ensure complete resection of the tumor, and supportive treatment was given after the operation, such as fluid replenishment, nutritional support, hepatoprotective drugs, and anti-infective drugs. The patient recovered well after the operation, and no recurrence has been observed up to now.

## Conclusion

Intrahepatic biliary cystadenoma is a rare cause of obstructive jaundice, and it has no specificity in clinical manifestations, laboratory examinations, and imaging examinations. The combined application of CT, MRI, ERCP, and other examinations is helpful for the diagnosis of the disease. Radical excision is the ideal treatment for intrahepatic biliary cystadenoma.

## Data availability statement

The original contributions presented in the study are included in the article/supplementary material. Further inquiries can be directed to the corresponding author.

## Ethics statement

The studies involving human participants were reviewed and approved by the Ethics Committee of Qilu Hospital of Shandong University. The patients/participants provided their written informed consent to participate in this study. Written informed consent was obtained from the individual(s) for the publication of any potentially identifiable images or data included in this article.

## Author contributions

K-SL evaluated the patient. Y-CL and A-DS initiated the case report, reviewed the literature, and drafted the manuscript. K-SL: concept and design, study supervision, and critical revision of the manuscript for important intellectual content. All authors contributed to the article and approved the submitted version.
